# Disruption of KPC-producing *Klebsiella pneumoniae* membrane via induction of oxidative stress by cinnamon bark (*Cinnamomum verum* J. Presl) essential oil

**DOI:** 10.1371/journal.pone.0214326

**Published:** 2019-04-02

**Authors:** Shun-Kai Yang, Khatijah Yusoff, Mokrish Ajat, Warren Thomas, Aisha Abushelaibi, Riaz Akseer, Swee-Hua Erin Lim, Kok-Song Lai

**Affiliations:** 1 Department of Cell and Molecular Biology, Faculty of Biotechnology and Biomolecular Sciences, Universiti Putra Malaysia, Serdang, Selangor, Malaysia; 2 Department of Microbiology, Faculty of Biotechnology and Biomolecular Sciences, Universiti Putra Malaysia, Serdang, Selangor, Malaysia; 3 Department of Veterinary Preclinical Sciences, Faculty of Veterinary Medicine, Universiti Putra Malaysia, Serdang, Selangor, Malaysia; 4 Perdana University-Royal College of Surgeons in Ireland, School of Medicine, Perdana University, Serdang, Selangor, Malaysia; 5 Health Sciences Division, Abu Dhabi Women’s College, Higher Colleges of Technology, Abu Dhabi, United Arab Emirates; Universite Paris-Sud, FRANCE

## Abstract

*Klebsiella pneumoniae* (KP) remains the most prevalent nosocomial pathogen and carries the *carbapenemase* (*KPC*) gene which confers resistance towards carbapenem. Thus, it is necessary to discover novel antimicrobials to address the issue of antimicrobial resistance in such pathogens. Natural products such as essential oils are a promising source due to their complex composition. Essential oils have been shown to be effective against pathogens, but the overall mechanisms have yet to be fully explained. Understanding the molecular mechanisms of essential oil towards KPC-KP cells would provide a deeper understanding of their potential use in clinical settings. Therefore, we aimed to investigate the mode of action of essential oil against KPC-KP cells from a proteomic perspective by comparing the overall proteome profile of KPC-KP cells treated with cinnamon bark (*Cinnamomum verum* J. Presl) essential oil (CBO) at their sub-inhibitory concentration of 0.08% (v/v). A total of 384 proteins were successfully identified from the non-treated cells, whereas only 242 proteins were identified from the CBO-treated cells. Proteins were then categorized based on their biological processes, cellular components and molecular function prior to pathway analysis. Pathway analysis showed that CBO induced oxidative stress in the KPC-KP cells as indicated by the abundance of oxidative stress regulator proteins such as glycyl radical cofactor, catalase peroxidase and DNA mismatch repair protein. Oxidative stress is likely to oxidize and disrupt the bacterial membrane as shown by the loss of major membrane proteins. Several genes selected for qRT-PCR analysis validated the proteomic profile and were congruent with the proteomic abundance profiles. In conclusion, KPC-KP cells exposed to CBO undergo oxidative stress that eventually disrupts the bacterial membrane possibly via interaction with the phospholipid bilayer. Interestingly, several pathways involved in the bacterial membrane repair system were also affected by oxidative stress, contributing to the loss of cells viability.

## Introduction

*Klebsiella* spp. are Gram-negative rod shaped bacteria that cause bacterial pneumonia with a high fatality rate if infection remains untreated in the clinical setting [1]. Globally, the vast majority of *Klebsiella* infections are hospital-acquired. Nosocomial *Klebsiella* infections are mainly caused by *Klebsiella pneumoniae*, the medically most important species of the genus which primarily attacks immune-compromised individuals who are hospitalized and suffer from severe underlying diseases such as diabetes mellitus, chronic pulmonary obstruction or even cancer. It is estimated that *Klebsiella* spp. cause 8% of all nosocomial bacterial infections in the United States and Europe, with 50.1% of these cases being caused by *Klebsiella pneumoniae* placing *Klebsiella* spp. among the eight most important infectious pathogens in hospitals [1]. In 1983, the first report of a plasmid-mediated extended spectrum beta-lactamases (*ESBLs*) capable of hydrolyzing extended-spectrum cephalosporins was discovered [2,3]. Carbapenems are one of the last lines of antibiotic treatment for severe drug-resistant bacterial infections, and are now the treatment of choice for serious infections caused by pathogens carrying the *ESBL* gene. This has led to an increased reliance on carbapenems in clinical practice [4]. In tandem with this, the first carbapenemase producing *K*. *pneumoniae* isolate was reported in North Carolina in 2001. This enzyme was termed *K*. *pneumoniae* carbapenemase (KPC) and conferred resistance to carbapenem antibiotics [5]. KPCs are encoded by the gene *bla*KPC, whose potential for inter-species and geographic dissemination is largely explained by its location within a Tn3-type transposon, Tn4401 that is capable of inserting itself into diverse plasmids of Gram-negative bacteria. Although *K*. *pneumoniae* remains the most prevalent bacterial species carrying KPCs, the enzyme has been identified in several other Gram-negative bacilli such as *Escherichia coli*, *Pseudomonas aeruginosa* and *Salmonella enterica* due to horizontal gene transfer [6]. To worsen this issue, KPC-producing *K*. *pneumoniae* (KPC-KP) possesses innate antibiotic resistance in the form of an efflux pump, which generally removes the antibiotics that have penetrated the bacterial membrane, from the cytoplasm into the extracellular environment. Membrane permeability can also be altered in the presence of antibiotics; preventing the access of antibiotics into the cells, which when coupled to the other mechanisms, enables resistance against higher concentrations of antibiotics [7].

In order to address to this particular issue, there had been constant efforts to discover novel antimicrobials for clinical use. Natural products such as essential oil consisting a plethora of chemical compounds, are becoming a popular mainstream platform for researchers in drug discovery [8]. Numerous studies have also demonstrated the efficacy of essential oils from curry plant (*Helichrysum italicum* (Roth) G. Don fil.), peppermint (*Mentha* x *piperita* L.), tea tree (*Melaleuca alternifolia* (Maiden & Betche) Cheel.) and marjoram (*Origanum majorana* L.) as promising antimicrobials. Multiple studies have shown the synergistic effects between various essential oils and antibiotics, potentially solving the antibiotic resistance issue in the clinical setting [9–14]. Despite this, only a few studies have been carried out to elucidate the mode of action of several essential oils on different bacteria; most of these studies have postulated that essential oils exert their antimicrobial activities by disrupting bacterial cell membrane and/or their efflux systems through various assays [15–17]. For example, de Souza et al. (2009) postulated that *Origanum vulgare* L. essential oil affects the membrane permeability of *Staphylococcus aureus* by studying potassium ion efflux and scanning electron microscopy [15]. Similarly, Silva et al. (2011) hypothesized that coriander essential oil exerts its bactericidal activity towards both Gram-positive and Gram–negative bacteria via membrane damage by measuring their efflux activity, respiratory activity and membrane potential [16]. To further support and understand the antimicrobial activity of essential oils, mass spectrometry-based proteomics analysis has become the tool of choice offering the identification and quantification of the proteome of an organism. There has been a tremendous improvement in instrument performance and the computational tools used in proteomic studies in recent years, which facilitates the understanding of the mechanisms of action of potential antimicrobial agents in the clinical setting. In the most widely used “bottom-up” approach to proteomics, liquid chromatography coupled with mass spectrometry (LC-MS/MS), enables a complex mixture of proteins to be first subjected to enzymatic cleavage; the resulting peptide products are separated based on chemical or physical properties and analyzed using a mass spectrometer. The proteome can then be analyzed, quantified and compared by using third party analytical software such as Progenesis QI (Progenesis Group Sdn. Bhd.) or Perseus (Max Planck Institute of Biochemistry). For instance, Xu et al. (2015) identified the mode of action of paclitaxel as chemotherapeutic drugs in HeLa cells by tampering with the abundance of tumor suppressor PDCD4 via LC-MS/MS proteomic profiling [18]. Similarly, Kawatani et al. (2016) also revealed the role of collismycin A as an iron chelator antagonizing cancer cells, using a proteomic approach [19].

In our previous study, we have successfully shown that cinnamon bark (*Cinnamomon verum* J. Presl) essential oil (CBO) is, in fact, an effective antimicrobial when used against KPC-KP with an extremely low minimum inhibitory concentration (MIC) of 0.16% (v/v) [11]. Furthermore, combinations of CBO and antibiotic meropenem further reduced the MIC of CBO to 0.08% (v/v) which makes it a putative candidate to be used as an antimicrobial in the clinical setting [11]. Other studies showed that CBO with a low MIC value is effective against a variety of Gram-positive and Gram–negative pathogens. Zamirah and colleagues (2013) demonstrated that CBO is effective against oral pathogens such as *Aggregatibacter actinomycetemcomitans*, *Fusobacterium nucleatum*, *Porphyromonas gingivalis*, *Streptococcus salivarius*, *S*. *mitis* and *S*. *mutans* with a low MIC ranging from 0.02 to 0.06% (v/v) [20]. Kaskatepe et al (2016) also showed that CBO is highly effective against other opportunistic pathogens, including *Escherichia coli*, *Pseudomonas aeruginosa* and multidrug-resistant *Staphylococcus aureus* (MRSA), with a MIC ranging from 0.009% to 0.078% (v/v) [21]. As already mentioned above, preliminary studies had postulated that essential oils affect bacterial membrane and/or their efflux system via numerous assays. Nevertheless, none had explained or gave an overview on the overall changes that the bacterial cells undergo when exposed to essential oils, especially in the perspective of proteomics. Thus, this study was carried out to understand and possibly bridge the missing links among previous studies regarding the mode of action of essential oils as an antimicrobial agent from the proteomic perspective, using CBO and KPC-KP as a model of study.

## Materials and methods

### CBO and KPC-producing *K*. *pneumoniae*

CBO (Cinnamon bark Sri Lanka, lot number: 6488) used throughout the study was purchased from Aroma Trading Ltd. (Milton Keynes, UK). The composition of CBO had been determined in our previous study via GC-MS [10]. The CBO was filter-sterilized using a 0.22 μm PES syringe membrane filter (Bioflow, Malaysia). KPC-producing *K*. *pneumoniae*, *K*. *pneumoniae* BAA-1705 (KPC-KP) was purchased from American Type Culture Collection (ATCC, Manassas, VA, USA) and was cultured on Mueller-Hinton broth (MHB) and agar (MHA) both from Sigma Aldrich, USA.

### CBO treatment and protein extraction

KPC-KP cell cultures were divided into two treatment groups, namely control (no treatment) and CBO treated prior to protein extraction. The CBO final concentration used was 0.08% (v/v), as determined by Yang et al. (2017) [11]. Both treatment groups had a final volume of 50 mL, supplemented with Tween 80 at final concentration of 10% to enhance the solubility of CBO and a standard inoculum of 1 × 10^5^ cfu/mL KPC-KP cells. Samples were incubated at 37°C with shaking at 200 rpm for 16 h to obtain sufficient cells for protein extraction. The cells from both treatment groups were pelleted by centrifugation at 9000 rpm for 10 min, washed for at least three times and resuspended in 500 μL cold protein extraction buffer (50 mM ammonium bicarbonate, 10 mM phenylmethylsulfonyl fluoride). Samples were then sonicated on ice at 20 amplitude for 10 cycles to lyse the cells; each cycle consisted of 10 seconds of sonication followed by 20 s of cooling, with a Qsonica Sonicator Q55 (Fischer Scientific, USA). Sonicated samples were then pelleted at 4°C and 10000 rpm for 1 h; supernatants were then collected and quantified via Bradford assay. The protein concentration of each sample was standardized to 1 mg/mL for the subsequent proteomic analysis. Treatment and analysis was standardized in three distinct biological replicates to ensure reproducibility of the experiment.

### Peptide digestion

Approximately 100 μg of total protein was resuspended in 100 μL of 50mM ammonium bicarbonate (pH 8.0). RapiGest (Waters Corporation, USA) at final concentration of 0.05% was added to the protein in equal parts. Protein from each sample was then concentrated to 100μL using Vivaspin column (GE Healthcare, USA) with a molecular weight cut-off (MWCO) of 3000 and incubated at 80°C for 15 min. The proteins were reduced in the presence of 5 mM dithiothreitol (DTT) at 60°C for 30 min, and then alkylated in the dark using 10 mM iodoacetamide at room temperature for 45 min. Proteolytic digestion was performed using Trypsin Gold (Promega, USA) at a ratio of 1:200 parts of protein, followed by incubation at 37°C overnight. Tryptic digestion and RapiGest activity were terminated by the addition of 1 μL concentrated trifluoroacetic acid (TFA) followed by the incubation of samples at 37°C for 20 min. The tryptic peptide solution of each sample was centrifuged at 14000 rpm for 20 min, and the resulting supernatants were collected in clean microcentrifuges tube kept at -80°C until subsequent analysis.

### Peptide separation and MS analysis

The nanoLC-MS/MS analysis was performed on an Orbitrap Fusion Tribrid mass spectrometer (Thermo Scientific, USA). The samples (2 μL containing 2 μg peptides) were injected and separated on an EASY-nLC 1000 (Dionex, Thermo Scientific, USA) equipped with an Easy-Spray Column Acclaim PepMap C18 100Å (2 μm, 50 μm × 15 cm, Thermo Scientific, USA). Samples were separated at a flow rate of 250 nL/min and using a gradient of 5% to 50% acetonitrile (ACN) in 0.1% formic acid (FA) for 45 min followed by a further gradient to 85% ACN in 0.1% FA for 2 min. The column was equilibrated back to 5% ACN with 0.1% FA over 1 min and maintained at 5% CAN until the next sample injection. Mass spectrometry was conducted in a positive ion mode with a nanospray voltage of 1.5 kV and a source temperature of 250°C. The instrument was operated in a data-dependent acquisition (DDA) mode with an Orbitrap MS (OTMS) survey scan using the following parameters: mass range of m/z 310–1800 with resolving power of 120000, automatic gain control (AGC) of 400000 and a maximum injection time of 50 ms. The Top Speed Mode of 3 seconds was used in the selection of precursors with the monoisotopic charge state of 2 to 7. These precursors were further analysed by MS/MS scanning. All precursors were filtered using a 20 s dynamic exclusion window and intensity threshold of 5000. The MS/MS spectra were analysed using ion trap MS (ITMS) with the following parameters: rapid scan rate with resolving power of 60000, AGC of 100, isolation window of 1.6 m/z and maximum injection time of 250 ms. Precursors were then fragmented by collision-induced dissociation (CID) and high-energy collision dissociation (HCD) at normalized collision energy of 30% to 28%.

### Protein identification

Raw data were processed using Thermo Scientific Proteome Discoverer Software v2.1 with the SEQUEST HT search engine. The MS ion intensities were calculated based on the accurate mass and time tag strategy. The accurate alignment of the detected LC retention time and *m/z* value across different analyses, together with the area under chromatographic elution profiles of the identified peptides could be compared between different samples. For protein identification, data was searched against the Uniprot *K*. *pneumoniae* database with 1% strict FDR and 5% relaxed FDR criteria using Percolator. Search parameters were set to 2 miscleavages with fixed modification of carbamidomethylation and variable modification through methionine oxidation or asparagine and glutamine deamidation. A fragment tolerance of 0.6 Da and a precursor tolerance of 10 ppm were used with trypsin as a digestion enzyme. Proteins with at least 2 unique peptides implied a greater confidence of protein identity.

### Protein quantification and data analysis

Protein quantification and statistical analyses were performed using Perseus Software v1.6.0.7 (Max Planck Institute of Biochemistry). Each control and treated samples analysis consisted of three biological replicates with three technical replicates, independently injected into the LCMS/MS. The protein file with three technical replicates in txt. format from Proteome Discoverer software were uploaded to Perseus for further comparative analysis between the samples. The data were log2-transformed to stabilise the variance and scale-normalised to the same mean intensity across the technical replicates. The mean values for all three technical replicates of the same biological samples were grouped together in the same matrix and valid values were obtained by filtering with ‘at least 2’, eliminating proteins which only existed in one of the technical replicates. Finally, all biological replicates of the same treatment group were consolidated into the same matrix, with the missing values imputated with the random number derived from a normal distribution. The histograms were plotted to compare the ratio distributions between all samples. Differentially expressed proteins between control and treatment were detected using a T-test, the *p*-values were also adjusted for multiple-testing using the permutation-based false discovery rate, with a number of randomization of 250. Proteins were considered to be significantly differentially expressed between treatment groups with adjusted *p*-values of <0.05 and a fold changes of ≤ -1 or ≥ +1.

### CBO treatment, RNA extraction and cDNA synthesis

KPC-KP cell culture were treated with CBO or vehicle buffer prior to RNA extraction. The CBO final concentration used was 0.08% (v/v), as determined in our previous work [11]. Both treatment groups had a final volume of 50 mL, and contained Tween 80 at final concentration of 10% to enhance the solubility of CBO. The standard KPC-KP cell inoculum was 1 × 10^5^ cfu/mL. Samples were incubated at 37°C with shaking at 200 rpm for 4 h followed by RNA extraction using the TransZol RNA purification kit (Transgen Biotech, China). RNA (0.5 ng) was subjected to reverse transcription with QuantiNova Reverse Transcription Kit (QIAGEN, Germany) in a 20-μl reaction volume. The synthesized complementary DNA (cDNA) was stored at −20 °C until further use.

### Proteomic expression validation through qRT-PCR analysis

The RNA abundance for several upregulated proteins from CBO-treated *K*. *pneumoniae* BAA-1705 was determined by qRT-PCR using QuantiNova SYBR Green PCR (QIAGEN, Germany) on CFX96 Touch Real-Time PCR Detection System (Bio-Rad Laboratories, Inc, USA). The Livak method was employed to assess the relative expression of three upregulated genes, namely cytidine deaminase (*cdd*), thiamine phosphate synthase (*thiE*) and uridine phosphorylase (*udp*), one down regulated gene, namely, 3-hydroxydecanoyl-[acyl-carrier-protein] dehydratase (*fabA*) and two housekeeping genes, namely *16S rRNA* and *OmpK36* porin. Reactions were performed in triplicate and data were analysed by using the CFX Manage Software (Bio-Rad). The thermal cycling conditions were as follows: 95 °C for 2 min, followed by 40 cycles of 95 °C for 5 s, and 60 °C for 10 s. In all experiments, no template reactions were used as negative controls.

## Results and discussion

### Comparative proteome profiling of KPC-KP treated with CBO

**Fig 1:**
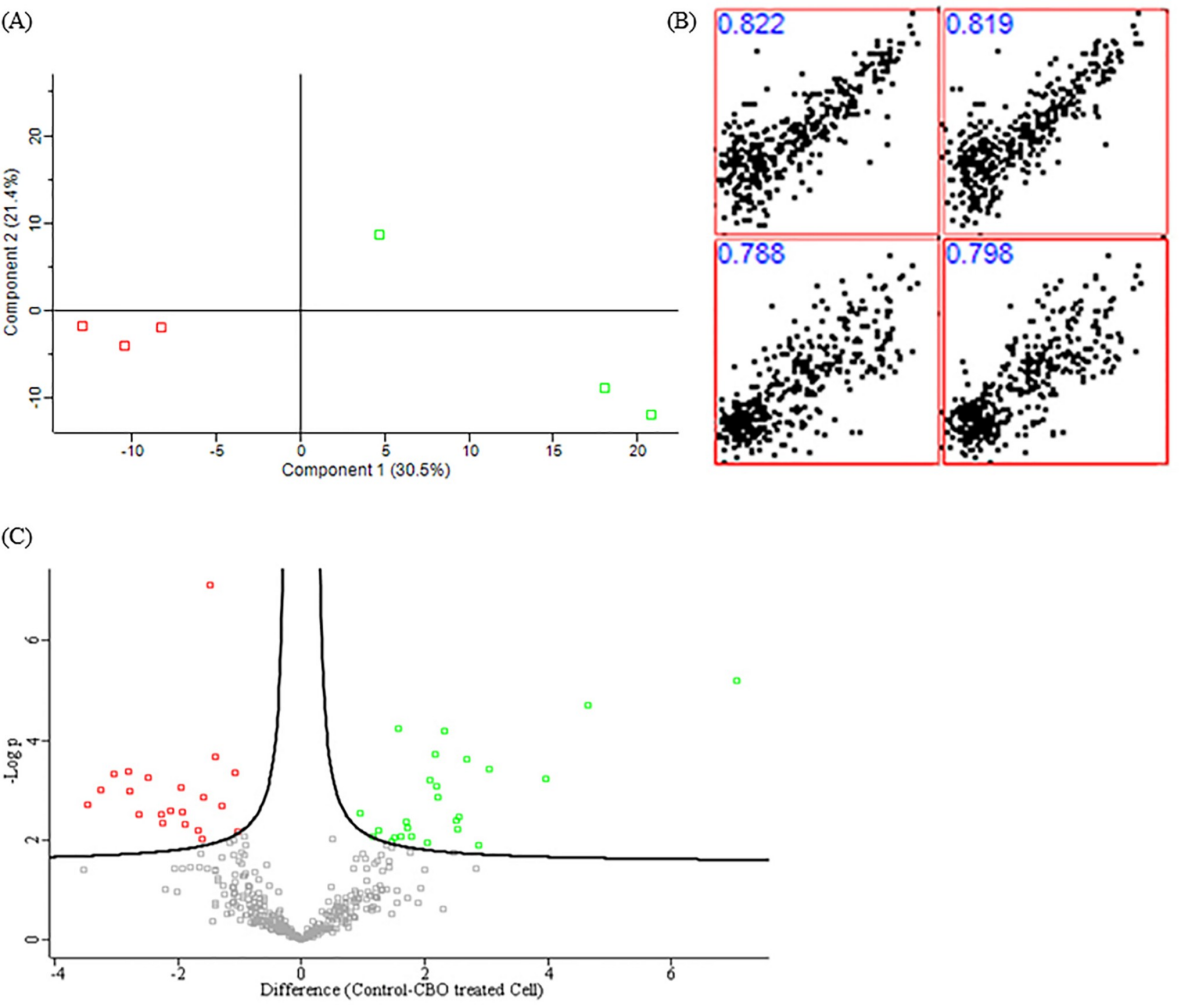
Exploratory analysis output of non-treated and CBO-treated KPC-KP cells using Perseus v1.6.0.7 software. (A) Principal component analysis (PCA) identifies differences between the non-treated (designated with green square) and CBO-treated (designated with red square) KPC-KP cells. (B) Multi-scatter plot with the Pearson correlation value of one of the profiles from the non-treated and CBO-treated KPC-KP cells. (C) Volcano plot showing up- (designated with green square) and downregulated (designated with red square) proteins from the CBO-treated KPC-KP cells.

Comparative proteomic analysis was carried out between non-treated and CBO-treated KPC-KP cells in four independent experiments using Perseus Software v1.6.0.7 (Max Planck Institute of Biochemistry). The protein profiles of treated and non-treated cells varied significantly with no outliers from the replicates, as shown in the principle component analysis (PCA) in Fig 1A. In addition, the Pearson correlation values between non-treated and CBO-treated KPC-KP were of high confidence (0.788 to 0.822), indicating that both the compared groups are of the same organism with no contamination within the samples (Fig 1B). The volcano plot (Fig 1C) of the comparative proteome between non-treated and CBO-treated KPC-KP cells showed a total of 46 proteins with significantly different abundance; 25 proteins of which were upregulated, whereas the other 21 proteins were downregulated in response to CBO treatment.

**Fig 2:**
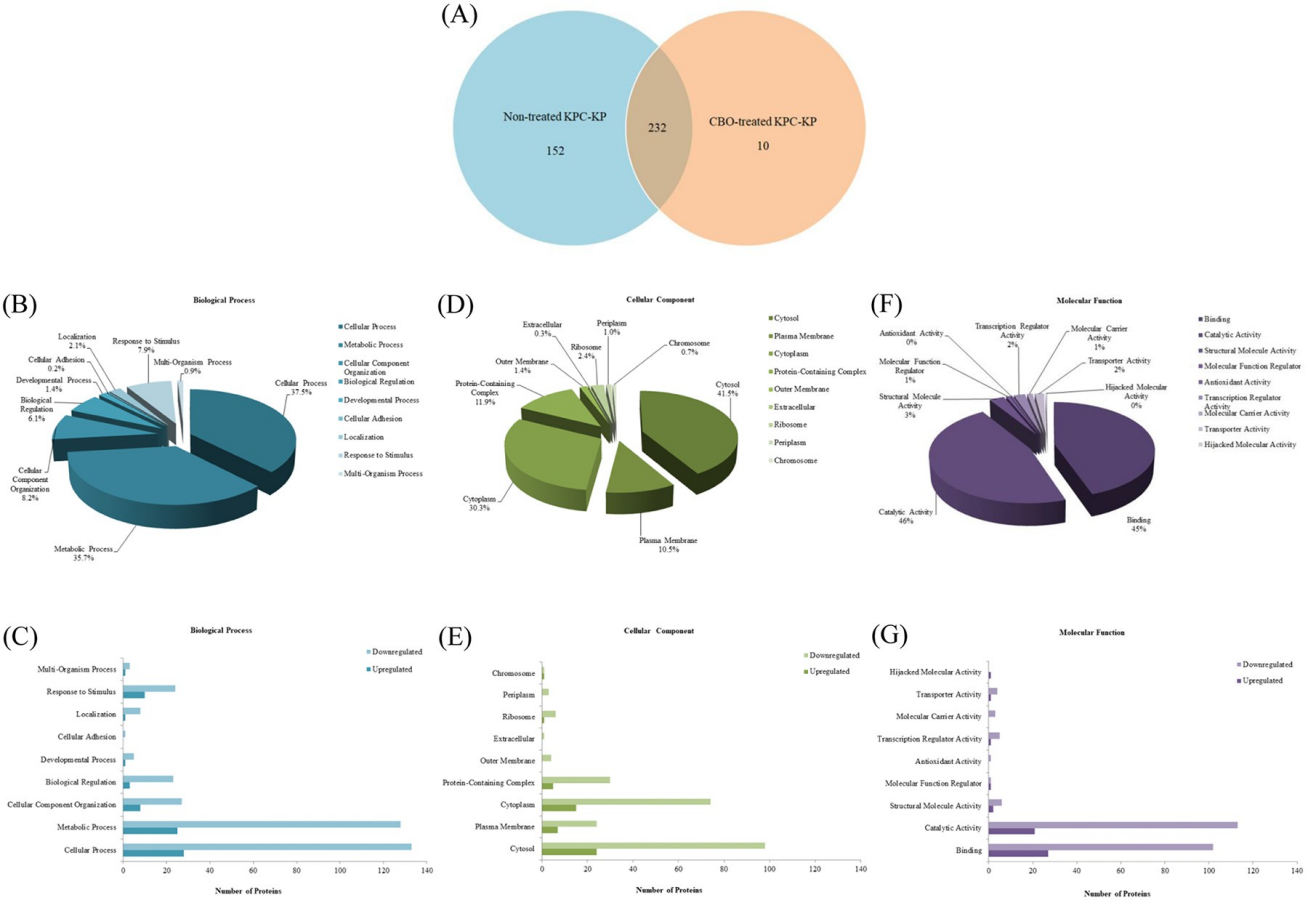
Comparative proteomic analysis of protein from CBO-treated KPC-KP cells. (A) Venn diagram of the total protein obtained from non-treated KPC-KP and CBO-treated KPC-KP cells, a total of 394 proteins were identified in non-treated KPC-KPs (384 proteins) and CBO-treated KPC-KPs (242 proteins) together. These total proteins were categorized based on the related biological process (B), cellular component (D) and molecular function (F). The numbers of upregulated and downregulated genes were also compared with respect to the relevant biological process (C), cellular component (E) and molecular function (G). Gene ontology analysis in terms of biological process of protein with significant abundance from CBO-treated KPC-KP cells.

A total of 384 proteins were identified from the non-treated cells, whereas only 242 proteins were identified from the CBO-treated cells, A total of 152 proteins were only expressed in the non-treated cells while 10 proteins were only expressed in the CBO-treated cells, the other 232 proteins were present in both groups of cells (Fig 2A). Proteins that were present in both samples were compared within the Perseus software to measure up- and downregulation of proteins while the rest of the proteins are referred as absent or present from each treatment group (Table 1). Proteins that were differentially present or absent, and up- or downregulated between the two groups of cells were subjected to gene ontology analysis with regards to biological process involvement, cellular component and molecular function (Fig 2). The majority of the proteins identified were linked to cellular and metabolic processes (37.5% and 35.7%), a number were also involved with cellular component organization and response to stimulus (8.2% and 7.9%) (Fig 2B). Cellular component wise, the majority of the proteins identified were categorized under cytosol and cytoplasm (41.5% and 30.3%) followed by macromolecular complex and plasma membrane (11.9% and 10.5%; Fig 2d). The categorization by molecular function identified proteins that were involved in catalytic activity and binding (46% and 45%) in addition to proteins that were structural molecules and or had transcription regulator activity (2.8% and 2.1%) (Fig 2F). Most proteins involved in biological processes, cellular component and molecular function were downregulated (Fig 2C, 2E and 2G).

**Table 1:** Top 100 proteins showing significant abundance difference (together with their accession numbers) in KPC-KP cells treated with CBO. The list is sorted according to descending positive or negative fold changes of each protein.

No	Proteins	Gene Name	Uniprot Accession No.	General Function	Fold Change
Upregulated Proteins					
1	Autonomous glycyl radical cofactor	grcA	A6TCJ1	Stress response	7.07
2	Beta-galactosidase 2	lacZ2	A6TI29	Carbohydrate metabolism	4.65
3	50S ribosomal protein L33	rpmG	B5XTG7	Protein biosynthesis	3.97
4	Sucrose porin	scrY	P27218	Transport	3.05
5	ATP-dependent protease ATPase subunit HslU	hslU	B5XZ37	Stress response	2.68
6	Uridine phosphorylase	udp	P52671	Pyrimidine biosynthesis	2.57
7	Agmatinase	speB	B5XUB2	Amine and polyamine biosynthesis	2.55
8	Phosphoribosylaminoimidazole-succinocarboxamide synthase	purC	A6TC99	Purine biosynthesis	2.52
9	Phosphomethylpyrimidine synthase	thiC	A6TGQ1	Thiamine biosynthesis	2.33
10	Phosphoribosylformylglycinamidine cyclo-ligase	purM	A6TCB3	Purine biosynthesis	2.21
11	NAD-dependent malic enzyme	maeA	A6T9K7	Carbohydrate metabolism	2.19
12	Probable Fe(2+)-trafficking protein	yggX	A6TDX0	Stress response	2.17
13	Thiamine-phosphate synthase	thiE	A6TGQ0	Thiamine biosynthesis	2.08
14	Chaperone protein DnaK	dnaK	A6T4F4	Stress response	1.73
15	Iron-sulfur cluster insertion protein ErpA	erpA	A6T4W0	Stress response	1.70
16	Cytidine deaminase	cdd	A6TBN1	Purine biosynthesis	1.62
17	Elongation factor Ts	tsf	A6T4X2	Protein biosynthesis	1.58
18	Ribosomal protein S12 methylthiotransferase RimO	rimO	A6T6T1	RNA processing and modification	1.50
19	Dual-specificity RNA methyltransferase RlmN	rlmN	A6TCD6	RNA processing and modification	1.26
20	Ribosomal RNA small subunit methyltransferase C	rsmC	A6THY6	RNA processing and modification	1.16
CBO-treated KPC-KP Exclusive Proteins					
21	Bifunctional protein GlmU	glmU	B5XZM7	Peptidoglycan biosynthesis	CBO-treated
22	Carbon storage regulator homolog	csrA	B5XVB9	Protein biosynthesis	CBO-treated
23	Cobalamin biosynthesis protein CobD	cobD	A6TDC6	Cobalamin biosynthesis	CBO-treated
24	DNA ligase	ligA	A6TC47	Stress response	CBO-treated
25	Ferrochelatase	hemH	B5Y0N2	Porphyrin biosynthesis	CBO-treated
26	Hydroxyacylglutathione hydrolase	gloB	A6T510	Secondary metabolite metabolism	CBO-treated
27	Integration host factor subunit beta	ihfB	A6T704	DNA processing	CBO-treated
28	Probable malate:quinone oxidoreductase (Fragment)	mqo	O32719	Energy synthesis	CBO-treated
29	tRNA 2-thiocytidine biosynthesis protein TtcA	ttcA	B5XRN3	RNA processing and modification	CBO-treated
Downregulated Proteins					
30	3-hydroxydecanoyl-[acyl-carrier-protein] dehydratase	fabA	A6T748	Fatty acid biosynthesis	-3.46
31	ATP-dependent protease subunit HslV	hslV	B5XZ36	Stress response	-3.25
32	HTH-type transcriptional regulator IscR	iscR	A6TCF2	Stress response	-3.05
33	Ribosome maturation factor RimP	rimP	A6TEI9	Ribosome biogenesis	-2.80
34	50S ribosomal protein L32	rpmF	A6T7E9	Translation	-2.78
35	Recombination-associated protein RdgC	rdgC	B5Y109	DNA processing	-2.48
36	Peptide methionine sulfoxide reductase MsrB	msrB	A6T7R0	Stress response	-2.27
37	6-phosphogluconate dehydrogenase, decarboxylating	gnd	P41576	Carbohydrate metabolism	-2.24
38	50S ribosomal protein L17	rplQ	A6TEU7	Protein biosynthesis	-2.13
39	Cysteine desulfurase IscS	iscS	A6TCF1	Stress response	-1.95
40	50S ribosomal protein L5	rplE	A6TEW0	Protein biosynthesis	-1.94
41	Uridine kinase	udk	A6TBG6	Pyrimidine biosynthesis	-1.89
42	Probable septum site-determining protein MinC	minC	A6TAX3	Cell division	-1.67
43	UDP-4-amino-4-deoxy-L-arabinose—oxoglutarate aminotransferase	arnB	A6TFA0	Lipopolysaccharide biosynthesis	-1.62
44	S-adenosylmethionine synthase	metK	A6TDV1	Protein biosynthesis	-1.58
45	Glutamate—tRNA ligase	gltX	A6TC43	Protein biosynthesis	-1.49
46	Arginine—tRNA ligase	argS	A6TB43	Protein biosynthesis	-1.39
47	HTH-type transcriptional regulator MalT	malT	A6TF41	Carbohydrate metabolism	-1.28
48	Protein TolB	tolB	B5XZC1	Cell division	-1.10
49	Acetyl-coenzyme A carboxylase carboxyl transferase subunit alpha	accA	A6T4Y7	Lipid metabolism	-1.08
50	30S ribosomal protein S3	rpsC	B5XNA0	Protein biosynthesis	-1.04
Control KPC-KP Exclusive Proteins					
51	10 kDa chaperonin	grosA	B5Y369	Stress response	Control
52	1-deoxy-D-xylulose-5-phosphate synthase	dxs	A6T5F3	Thiamine biosynthesis	Control
53	3-dehydroquinate synthase	aroB	A6TF13	Chorismate biosynthesis	Control
54	3-methyl-2-oxobutanoate hydroxymethyltransferase	panB	A6T4T0	Pantothenate biosynthesis	Control
55	3-octaprenyl-4-hydroxybenzoate carboxy-lyase	ubiD	A6TGM1	Ubiquinone biosynthesis	Control
56	3-phosphoshikimate 1-carboxyvinyltransferase	aroA	B5XY87	Chorismate biosynthesis	Control
57	4-hydroxy-3-methylbut-2-enyl diphosphate reductase	ispH	A6T4G3	Isoprenoid biosynthesis	Control
58	50S ribosomal protein L34	rpmH	A6TG05	Protein biosynthesis	Control
59	6-phosphogluconolactonase	pgl	A6T6J6	Carbohydrate metabolism	Control
60	Adenine phosphoribosyltransferase	apt	A6T5N2	Purine metabolism	Control
61	Aminomethyltransferase	gcvT	A6TDR7	Protein biosynthesis	Control
62	Argininosuccinate synthase	argG	A6TEJ0	Protein biosynthesis	Control
63	ATP synthase epsilon chain	atpC	A6TG35	Energy synthesis	Control
64	ATP-dependent RNA helicase RhlB	rhlB	A6TGG9	RNA processing and modification	Control
65	Biosynthetic arginine decarboxylase	speA	B5XUB1	Amine and polyamine biosynthesis	Control
66	Catalase-peroxidase	katG	A6T9H9	Stress response	Control
67	Cell division protein ZipA	zipA	A6TC48	Cell division	Control
68	Cell division topological specificity factor	minE	A6TAX5	Cell division	Control
69	Chorismate synthase	aroC	A6TC15	Chorismate biosynthesis	Control
70	Chromosomal replication initiator protein DnaA	dnaA	B5XT51	DNA processing	Control
71	D-amino acid dehydrogenase	dadA	A6TAW4	Protein biosynthesis	Control
72	Dihydroorotase	pyrC	A6T7D6	Pyrimidine biosynthesis	Control
73	Dihydroorotate dehydrogenase (quinone)	pyrD	A6T739	Pyrimidine biosynthesis	Control
74	Dihydroxy-acid dehydratase	ilvD	A6TGF8	Protein biosynthesis	Control
75	DNA gyrase inhibitor YacG	yacG	A6T4P3	DNA processing	Control
76	DNA mismatch repair protein MutS	mutS	A6TD24	Stress response	Control
77	DnaA initiator-associating protein DiaA	diaA	A6TEG8	DNA processing	Control
78	Electron transport complex subunit C	rnfC	B5XWP9	Energy synthesis	Control
79	Elongation factor P—(R)-beta-lysine ligase	epmA	A6TH74	Protein biosynthesis	Control
80	Endonuclease V	nfi	A6TGQ5	Stress response	Control
81	Exodeoxyribonuclease 7 small subunit	xseB	B5Y0W9	DNA processing	Control
82	Formate-dependent phosphoribosylglycinamide formyltransferase	purT	B5XQ21	Purine metabolism	Control
83	Fructose-6-phosphate aldolase	fsa	A6TGD7	Carbohydrate metabolism	Control
84	Gamma-glutamyl phosphate reductase	proA	A6T561	Protein biosynthesis	Control
85	Glucans biosynthesis protein D	mdoD	B5XWS2	Glycan metabolism	Control
86	Glucokinase	glk	A6TC33	Energy synthesis	Control
87	Glucosamine-6-phosphate deaminase	nagB	A6T6C1	Carbohydrate metabolism	Control
88	Glutamate 5-kinase	proB	A6T562	Protein biosynthesis	Control
89	Glycerol-3-phosphate acyltransferase	plsB	A6TGV0	Phospholipid biosynthesis	Control
90	Histidine biosynthesis bifunctional protein HisB	hisB	A6TBC5	Protein biosynthesis	Control
91	Holliday junction ATP-dependent DNA helicase RuvA	ruvA	B5XQ04	Stress response	Control
92	HTH-type transcriptional regulator CysB	cysB	P45600	Protein biosynthesis	Control
93	HTH-type transcriptional repressor PurR	purR	A6TA06	Purine metabolism	Control
94	Hydroxyethylthiazole kinase	thiM	A6TBJ8	Thiamine biosynthesis	Control
95	Imidazole glycerol phosphate synthase subunit HisF	hisF	A6TBC8	Protein biosynthesis	Control
96	Iron-binding protein IscA	iscA	A6TCE9	Stress response	Control
97	Ketol-acid reductoisomerase (NADP(+))	ilvC	A6TGG1	Protein biosynthesis	Control
98	Large-conductance mechanosensitive channel	mscL	B5XNC0	Transport	Control
99	L-lactate dehydrogenase	lldD	A6TFK0	Energy synthesis	Control
100	L-threonine 3-dehydrogenase	tdh	A6TFL2	Protein biosynthesis	Control

Of the overlapping 232 proteins identified in both treatment groups, only 41 proteins showed significant differences, in terms of fold change between the groups. The majority of the proteins which showed significant abundance difference were downregulated (51.2%) when compared to the non-treated KPC-KP cells, whereas the other 48.8% of the proteins were upregulated as shown in Table 1. Proteins that were absent or present in each treatment group were also listed in Table 1, with proteins that were only present in the CBO-treated KPC-KP cells listed under the upregulated proteins whereas proteins which were only present in the non-treated KPC-KP cells listed under the downregulated protein section. These proteins were then subjected to KEGG pathway analysis to elucidate mechanism involved in the action of CBO on KPC-KP cells.

### Bacterial membrane disruption

The Gram negative bacterial cell outer barrier consists of three separate component, the outer membrane, the peptidoglycan and the plasma membrane [22]. Both the outer membrane and the plasma membrane are made up of a phospholipid bilayer embedded with membrane proteins. The biochemical feature which differentiates the layers is the presence of lipopolysaccharides uniquely in the outer membrane. Of the identified proteins from the KPC-KP cells, 10.5% were located at the bacterial membrane (Fig 2D and 2E). Following exposure to CBO, our proteomic profiling showed 5 outer membrane exclusive proteins and 26 plasma membrane exclusive proteins were completely lost after the exposure to CBO (Table 2). Similarly, Wu and colleagues (2016) found that 3-*p*-trans-coumaroyl-2-hydroxyquinic acid, a phenolic compound isolated from Himalayan cedar essential oil caused bacterial membrane damage and the loss of membrane proteins due to specific interactions between the compound and the lipid and proteins within the bacterial membrane [23]. This disrupted the membrane integrity of the bacteria which caused the loss of plasma membrane protein. The downregulation of outer membrane integrity regulators such as the TolB porin-interacting protein, the large-conductance mechanosensitive channel and outer membrane protein assembly factor BamA in KPC-KP cells exposed to CBO also indicated the loss of membrane integrity in KPC-KP cells [24]. In addition, proteins involved in energy generation, such as the ATP synthase, the electron transport complex and the NADH-quinone oxidoreductases which are often embedded within the bacterial membrane were lost completely. This is yet another indicator for disrupted bacterial membrane integrity that may contribute to bacterial cell killing through the shutdown ofenergy production in CBO-treated KPC-KP cells. With compromised plasma membrane integrity, intracellular proteins easily escape into the extracellular environment as suggested by Ukuku et al. (2007) [25].

**Table 2:** List of proteins belonging to the outer membrane and plasma membrane of KPC-KP cells and their relative status in proteomic profiling.

Uniprot Accession No.	Protein	Status
Outer membrane protein		
A6TGU6	Maltoporin 2	Lost
P40786	Nucleoside-specific channel-forming protein tsx	Lost
P24017	Outer membrane protein A	Upregulated by 2.04 folds
A6T4X9	Outer membrane protein assembly factor BamA	Lost
A6T7G4	Penicillin-binding protein activator LpoB	Lost
P27218	Sucrose porin	Lost
Plasma membrane protein		
A6TGM1	3-octaprenyl-4-hydroxybenzoate carboxy-lyase	Lost
A6TG35	ATP synthase epsilon chain	Lost
B5XZ37	ATP-dependent protease ATPase subunit HslU	Lost
B5XUB1	Biosynthetic arginine decarboxylase	Lost
A6TC48	Cell division protein ZipA	Lost
A6TAX5	Cell division topological specificity factor	Lost
A6T4F4	Chaperone protein DnaK	Upregulated by 1.73 folds
B5XT51	Chromosomal replication initiator protein DnaA	Lost
A6TDC6	Cobalamin biosynthesis protein CobD	Upregulated*
A6TAW4	D-amino acid dehydrogenase	Lost
A6T739	Dihydroorotate dehydrogenase (quinone)	Lost
B5XWP9	Electron transport complex subunit C	Lost
B5XWS2	Glucans biosynthesis protein D	Lost
A6TGV0	Glycerol-3-phosphate acyltransferase	Lost
A6TDR6	Glycine cleavage system H protein	Lost
B5XNC0	Large-conductance mechanosensitive channel	Lost
A6TFK0	L-lactate dehydrogenase	Lost
A6TEQ3	Malate dehydrogenase	Lost
B5XXP0	NAD(P)H dehydrogenase (quinone)	Lost
A6TBX3	NADH-quinone oxidoreductase subunit B	Lost
A6TBX2	NADH-quinone oxidoreductase subunit C/D	Lost
A6TC99	Phosphoribosylaminoimidazole-succinocarboxamide synthase	Upregulated by 2.52 folds
Q07411	Polyphosphate kinase	Lost
O32719	Probable malate:quinone oxidoreductase (Fragment)	Upregulated*
A6TDG4	Prolipoprotein diacylglyceryl transferase	Lost
P27219	PTS system sucrose-specific EIIBC component	Lost
A6TC94	Succinyl-diaminopimelate desuccinylase	Lost
A6THZ7	Thymidine phosphorylase	Lost
A6TGL3	Ubiquinone/menaquinone biosynthesis C-methyltransferase UbiE	Lost
A6T4N3	UDP-N-acetylglucosamine—N-acetylmuramyl-(pentapeptide) pyrophosphoryl-undecaprenol N-acetylglucosamine transferase	Lost

*refers to the proteins that were only present in CBO-treated KPC-KP cells.

### Oxidative stress

CBO contains a plethora of different chemical compounds which are dominated by a large group of oxygenated terpenes and terpenoids [10]. These compounds are hypothesized to be responsible for CBO’s action on the bacterial membrane. From our proteomic data, upregulation of proteins such as autonomous glycyl radical cofactor and catalase peroxidase in the CBO-treated KPC-KP cells suggest the presence of significant oxidative stress. Autonomous glycyl radical cofactor is upregulated by 7.07 fold, and acts as a radical domain which protects pyruvate formate lyase from oxidative stress. Wagner et al. (2001) and Shisler et al. (2014) found that upregulation of glycyl radical cofactor indicates oxidative stress which affects the pyruvate formate lyase that is involved in glucose metabolism [26, 27]. In addition, catalase peroxidase, an enzyme which alleviates oxidative stress from reactive oxygen species (ROS), was also induced when KPC-KP cells were exposed to CBO. Under condition of oxidative stress, high abundance ROS cause oxidative damage to nucleic acids [28]. The detection of the DNA mismatch repair protein MutS and the DNA ligase following the exposure to CBO showed that genetic materials of KPC-KP had been damaged, and that elevated expression of these proteins could alleviate some of the oxidative DNA damage. The work of Vogel et al. (2011) showed that oxidative stress leads to the degradation of ribosomal protein [29] and this appears to be in line with our findings in CBO-treated KPC-KP cells where 14 ribosomal proteins and ribosome related proteins showed decreased abundance. Specifically, 30S ribosomal protein S3; 50S ribosomal proteins L17, L32, L34 and L5; ribosomal protein L11 methyltransferase; ribosomal RNA large subunit methyltransferase E, F and I; ribosomal RNA small subunit methyltransferase B and G; ribosome maturation factor M and P, and ribosome binding factor A were all reduced in abundance. As one of the key proteins in the maturation of 30S ribosomal subunit, ribosome maturation factor RimP protein is crucial in the process of protein translation by allowing correct pairing of the mRNA and the corresponding anticodon of the tRNA [30, 31]. In agreement with this finding is the decrease in abundance of the other three 50S ribosomal subunit fragments namely, L5, L17 and L32. This further supports the idea that induction of oxidative stress by CBO affects the abundance of ribosomal subunit fragments, as a result of the denaturation of protein fragments or the interruption of protein synthesis in KPC-KP cells. Cosentino et al. (2014) found that bergamot essential oil induced the production of ROS in polymorphonuclear leukocytes, contributing to enhanced eradication of infection [32]. Another study by Yoo et al (2005) also suggest that eugenol, one of the main constituents of essential oils such as from nutmeg and cinnamon bark induces the production of ROS in leukemia cells, eventually killing the cells by initiating apoptosis [33]. The induction of oxidative stress by essential oils seems to be in conflict with the perception that essential oils contain high concentration of antioxidants. We previously found that the CBO used in this study comprised 13 compounds, of which nine were antioxidants whereas the other four were not [10, 34–38]. These non-antioxidant compounds may be responsible for inducing the oxidative stress observed, by generating ROS or inhibiting anti-oxidizing systems [39]. Mimica-Dukić et al. (2016) suggested that essential oils may act both as antioxidants and also as pro-oxidant due to their complex chemistry [40]. Additionally, a single compound might exhibit dual antioxidant and prooxidant effects. For instance, Bezerra et al. (2017) found that eugenol had such dual effects, acting as an antioxidant in free-radical scavenging while inducing DNA damage via ROS generation [41]. So, the prooxidant activity within CBO may contribute to the oxidative stress, leading to lipid peroxidation in the cell membrane, while inhibiting the antioxidant activity, as indicated in the proteomic profile.

### KEGG pathway analysis

When analysing significant pathways via the Panther classification system we found that the main pathways associated with the proteins identified included the lipid, cell wall and lipopolysaccharide biosynthesis pathways.

#### Lipid biosynthesis

Lipids have critical functions in the bacterial cytoplasmic and outer membranes in separating the bacterial cytoplasm from the external environment [42]. KEGG pathway analysis identified 4 proteins which are involved in the lipid biosynthesis pathway that were affected by CBO treatment. Two of these proteins were downregulated in CBO-treated KPC-KP cells: 3-hydroxydecanoyl-[acyl-carrier-protein] dehydratase (-3.46 fold) and acetyl-coenzyme A carboxylase carboxyl transferase subunit alpha (-1.08 fold). While two other proteins were undetectable following CBO treatment: glycerol-3-phosphate acyltransferase and large-conductance mechanosensitive channel protein. The former two proteins are key components in the fatty acid biosynthesis pathway of Gram-negative bacteria. Acetyl-coenzyme A carboxylase carboxyl transferase is one of the major enzymes in the synthesis of malonyl-CoA, a substrate required in the synthesis of fatty acids [43]. 3-hydroxydecanoyl-[acyl-carrier-protein] dehydratase functions as an essential mediator in the synthesis of phospholipids in bacteria [44, 45]. As shown in Table 1, glycerol-3-phosphate acyltransferase (G3PAT) was undetectable in the CBO-treated KPC-KP cells. G3PAT, a rate determining enzyme belonging to the glycerolphospholipid metabolism pathway catalyzes the synthesis of phosphatidic acid from glycerol-3-phosphate and long-chain acyl-CoA, is an essential precursor in the synthesis of the bacterial phospholipid bilayer [46, 47]. The absence of G3PAT indicates a perturbed bacterial membrane repair system. This further indicates a disruption in the integrity of the phospholipid membrane in CBO-treated KPC-KP cells. The absence of the large-conductance mechanosensitive channel protein in the treated group also indicated a disrupted membrane structure as this channel regulates membrane stretching and stability under osmotic stress [48, 49]. Upon exposure to CBO, oxidative stress may promote protein denaturation and so affect bacterial cell membrane stretch capacity under osmotic pressure, eventually leading to increased CBO influx and bacterial cell killing.

#### Cell wall biosynthesis

Peptidoglycan in the cell wall is a major structural component in prokaryotic cells, forming a stable layer which protects the bacteria from lysis under osmotic stress [50]. The proteomic profile showed that three essential proteins in bacterial cell wall synthesis were lost upon exposure to CBO. This may indicate that CBO inhibited the expression of these proteins or that CBO-induced oxidative stress resulted in degradation of these proteins, so preventing cell wall synthesis and repair, and eventually causing cell death. These proteins included UDP-N-acetylglucosamine—N-acetylmuramyl-(pentapeptide) pyrophosphoryl-undecaprenol N-acetylglucosamine transferase (murG); penicillin-binding protein activator LpoB and succinyl-diaminopimelate desuccinylase. The MurG protein is involved in the biosynthesis of the N-acetylmuramic acid-N-acetylglucosamine intermediate, which form a single unit of peptidoglycan cell wall. In the absence of murG, no N-acetylmuramic acid would not be linked to N-acetylglucosamine, preventing the formation of peptidoglycan bilayer linkage and eventually killing the cell due to osmotic pressure and oxidative stress [50]. Additionally, succinyl-diaminopimelate desuccinylase also play a major role in maintaining the structure and integrity of the peptidoglycan, as this protein is essential in the synthesis of meso-diaminopimelic acid which is one of the penta-peptides found linked to N-acetylmuramic acid, and is crucial for the cross-linkage with the opposite layer of N-acetylglucosamine. Interestingly, penicillin-binding protein activator LpoB which stimulates the biosynthesis of peptidoglycan molecules was not detected in CBO treated KPC-KP cells. These findings may indicate that the peptidoglycan biosynthesis and repairing had been completely shut down [51].

#### Lipopolysaccharide biosynthesis

Lipopolysaccharide (LPS) is a highly acylated saccharolipid located on the outer layer of the outer membrane of Gram-negative bacteria. LPS is crucial in the maintenance of membrane integrity and has a barrier function which prevents the passive diffusion of hydrophobic solutes, such as antibiotics and detergents into the cell [52]. Many studies have postulated that CBO exerts its bactericidal activity through damage to the outer membrane which allows other bactericidal molecules to enter the cell, eventually killing the cells [11, 15, 16]. LPS consist of a few components including lipid A; core oligosaccharide and O-antigen [53]. We identified three proteins that are involved in LPS biosynthesis that became undetectable upon CBO exposure. These were O-antigen export system ATP-binding protein (RfbB); UDP-4-amino-4-deoxy-L-arabinose—oxoglutarate aminotransferase (arnB) and UDP-3-O-acyl-N-acetylglucosamine deacetylase (lpxC). In order to achieve resistance towards antibiotics such as polymyxin, the 4-amino-4-deoxy-L-arabinose moiety must be added to the lipid A [54]. This reaction is catalyzed by the arnB protein which is not detected in the KPC-KP cells exposed to CBO. Furthermore, lpxC protein, a critical enzyme which catalyzes the synthesis of lipid A was also undetectable in CBO-treated KPC-KP cells. 90% of the bacterial outer membrane consists of lipid A, which is crucial for the attachment of core oligosaccharide as well as o-antigen, granting antibiotic resistance to bacteria while maintaining the membrane integrity. In the absence of the lpxC protein, the outer membrane integrity cannot be maintained, compromising cellular resistance against antibiotics. Due to the importance of this protein in lipid A biosynthesis, lpxC has been a target for the development of novel antimicrobial drugs [55, 56]. The O-antigen export system ATP-binding protein, which facilitates the export of O-antigen into the outer membrane was also not detected following exposure to CBO. Thus, CBO may contain compounds that inhibit the expression of these important membrane proteins and sensitize bacteria to antibiotics and changes in osmotic pressure.

### qRT-PCR analysis of differentially expressed proteins

#### Standard curve for qRT-PCR

We selected four genes for further validation of our proteomic data using qRT-PCR together with two housekeeping genes, namely *3-hydroxydecanoyl-[acyl-carrier-protein] dehydratase* (*fabA*), *cytidine deaminase* (*cdd*), *thiamine phosphate synthase* (*thiE*), *uridine phosphorylas*e (*udp*), *16s rRNA* and *OmpK36 porin*. Out of these, three were significantly higher in abundance and one was less abundant in the proteomic profile following exposure to CBO. The designed primers for each selected genes were listed in S1 Table. Their efficiency ranged within 90 and 100%.

#### Relative expression level of selected genes

**Fig 3:**
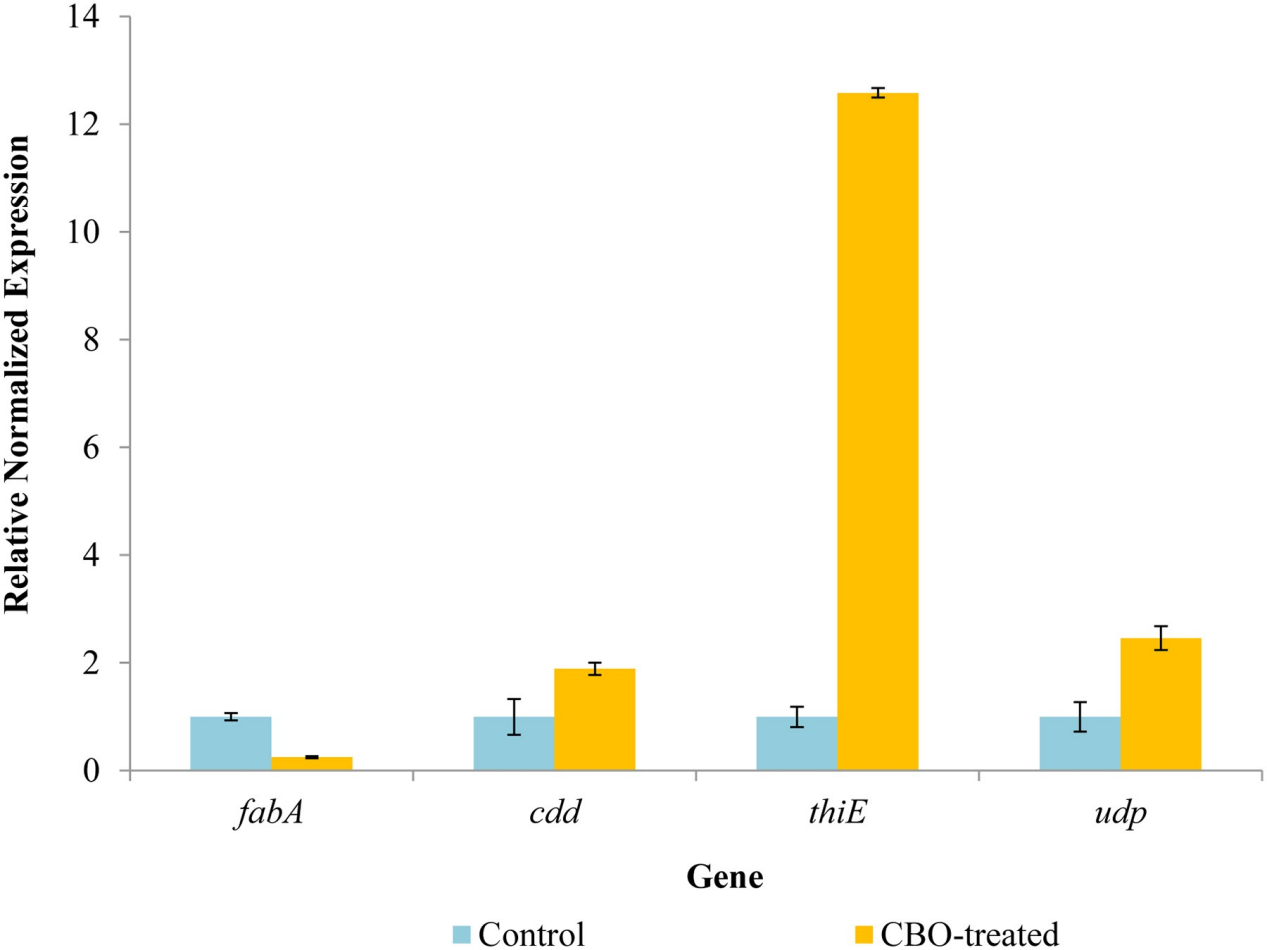
Expression patterns of *fabA*, *cdd*, *thiE* and *udp* genes in KPC-KP cells subjected to CBO treatment. Results are presented as differential relative transcript abundance.

The relative abundance of mRNA for the differentially expressed proteins investigated was measured by qRT-PCR, comparing the untreated cells with CBO-treated KPC-KP cells (Fig 3). Proteomic profiling found that *fabA* was downregulated by -3.46 fold, whereas *cdd*, *thiC* and *udp* were upregulated by 1.62, 2.08 and 2.57 fold respectively (Table 1). The qRT-PCR analysis of mRNA abundance found that *fabA* was downregulated by 0.8 fold whereas *cdd*, *thiC* and *udp* were upregulated by 0.8, 11 and 1.4 fold respectively. The pattern in mRNA abundance change following CBO treatment mirrors that of the protein abundance for these four gene products, indicating that at least part of the change in the abundance for these four proteins is due to changes in gene transcription. The differences in the magnitude of the fold changes between the protein abundance and mRNA transcript abundance may be a consequence of oxidative stress-induced protein degradation or different stabilities for the mRNA species. Nevertheless, the trends in expression changes shown in the protein profiles and mRNA transcription levels are consistent with each other and validate our results obtained from proteomic profiling.

CBO exerts antimicrobial activity on KPC-KP cells through membrane disruption, and proteomic profiling shows that the membrane damage induced was due to oxidative stress. This is supported by the increased in abundance of oxidative stress regulators when KPC-KP cells were exposed to CBO. A review by Itri et al. (2014) consolidated information from studies involving oxidative stress and membrane damage. The study concluded that oxidative stress would reduce the permeability and integrity of the plasma membrane, leading to leakage of intracellular contents and eventually killing the bacterial cells [57]. The membrane disruptive effects of CBO could also be deduced from our proteomic profiles, and numerous proteins showed decreased abundance or were lost following CBO exposure. Interestingly, CBO treatment also interfered with the biosynthesis of the plasma membrane, cell wall and outer membrane, disabling the structural repair system. The proteomic profiles were validated using qRT-PCR analysis, where proteomic changes were mirrored by changes in corresponding mRNA abundance. Together with the evidence from our previous study on the antimicrobial potential and the mode of action of CBO against KPC-KP cells [11], we showed that the antibacterial activity of CBO derives from its ability to induce oxidative stress in bacterial cells. The resulting oxidation disrupts the bacterial membrane, eventually enabling the influx of ROS into the cells and, at the same time, leads to intracellular content leakage. ROS induce genetic damage and impair DNA and membrane repair systems. Our results demonstrate that CBO causes oxidative stress, damage to bacterial membranes, cellular leakage and cell killing.

## Supporting information

S1 Attachment Raw data of non-treated and CBO-treated KPC-KP proteome profile. (ZIP)

S1 Dataset Comparative proteomic analysis spreadsheet. (XLSX)

S1 Table List of primers used in this study. (DOCX)

## References

[1] Durdu B, Hakyemez IN, Bolukcu S, Okay G, Gultepe B, Aslan T. Mortality markers in nosocomial *Klebsiella pneumoniae* bloodstream infection. Springerplus. 2016;5(1): 1892 10.1186/s40064-016-3580-8 27843749 PMC5084144

[2] Paterson DL, Bonomo RA. Extended-spectrum beta-lactamases: a clinical update. Clin Microbiol Rev. 2005;18(4): 657–686. 10.1128/CMR.18.4.657-686.2005 16223952 PMC1265908

[3] Ur Rahman S, Ali T, Ali I, Khan NA, Han B, Gao J. The Growing genetic and functional diversity of extended spectrum beta-lactamases. Biomed Res Int. 2018;2018:9519718 10.1155/2018/9519718 29780833 PMC5892270

[4] Rhomberg PR, Jones RN. Summary trends for the Meropenem Yearly Susceptibility Test Information Collection Program: a 10-year experience in the United States (1999–2008). Diagn Microbiol Infect Dis. 2009;65(4): 414–426. 10.1016/j.diagmicrobio.2009.08.020 19833471

[5] Yigit H, Queenan AM, Anderson GJ, Domenech-Sanchez A, Biddle JW, Steward CD, et al Novel carbapenem-hydrolyzing beta-lactamase, KPC-1, from a carbapenem-resistant strain of *Klebsiella pneumoniae*. Antimicrob Agents Chemother. 2001;45(4): 1151–1161. 10.1128/AAC.45.4.1151-1161.2001 11257029 PMC90438

[6] Kitchel B, Rasheed JK, Patel JB, Srinivasan A, Navon-Venezia S, Carmeli Y, et al Molecular epidemiology of KPC-producing *Klebsiella pneumoniae* isolates in the United States: clonal expansion of multilocus sequence type 258. Antimicrob Agents Chemother. 2009;53(8): 3365–3370. 10.1128/AAC.00126-09 19506063 PMC2715580

[7] Yang SK, Low LY, Yap PSX, Yusoff, Mai CW, Lai KS, et al Plant-derived antimicrobials: insights into mitigation of antimicrobial resistance. Rec Nat Prod. 2018;12(4): 295–316.

[8] Yap PSX, Yiap BC, Ping HC, Lim SHE. Essential oils, a new horizon in combating bacterial antibiotic resistance. Open Microbiol J. 2014;8: 6–14. 10.2174/1874285801408010006 24627729 PMC3950955

[9] Lorenzi V, Muselli A, Bernardini AF, Berti L, Pages JM, Amaral L, et al Geraniol restores antibiotic activities against multidrug-resistant isolates from gram-negative species. Antimicrob Agents Chemother. 2009;53(5): 2209–2211. 10.1128/AAC.00919-08 19258278 PMC2681508

[10] Yap PSX, Krishnan T, Chan KG, Lim SHE. Antibacterial mode of action of *Cinnamomum verum* bark essential oil, alone and in combination with piperacillin, against a multidrug-resistant *Escherichia coli* strain. J Microbiol Biotechnol. 2015;25(8): 1299–1306. 10.4014/jmb.1407.07054 25381741

[11] Yang SK, Yusoff K, Mai CW, Lim WM, Yap WS, Lim SHE, et al Additivity vs synergism: investigation of the additive interaction of cinnamon bark oil and meropenem in combinatory therapy. Molecules. 2017;22(11). 10.3390/molecules22111733 PMC6150308 29113046

[12] Yap PSX, Lim SHE, Hu CP, Yiap BC. Combination of essential oils and antibiotics reduce antibiotic resistance in plasmid-conferred multidrug resistant bacteria. Phytomedicine. 2013;20(8–9): 710–713. 10.1016/j.phymed.2013.02.013 23537749

[13] Yap PSX, Yang SK, Lai KS, Lim SHE. Essential oils: the ultimate solution to antimicrobial resistance in Escherichia coli. 2017.

[14] Yang SK, Yap PSX, Krishnan T, Yusoff K, Chan KG, Yap WS, et al Mode of action: synergistic interaction of peppermint (*Mentha x piperita* L. Carl) essential oil and meropenem against plasmid-mediated resistant *E*. *coli*. Rec Nat Prod. 2018;12(6): 13.

[15] de Souza EL, de Barros JC, de Oliveira CE, da Conceicao ML. Influence of *Origanum vulgare* L. essential oil on enterotoxin production, membrane permeability and surface characteristics of *Staphylococcus aureus*. Int J Food Microbiol. 2010;137(2–3): 308–311. 10.1016/j.ijfoodmicro.2009.11.025 20015563

[16] Silva F, Ferreira S, Queiroz JA, Domingues FC. Coriander (*Coriandrum sativum* L.) essential oil: its antibacterial activity and mode of action evaluated by flow cytometry. J Med Microbiol. 2011;60: 1479–1486. 10.1099/jmm.0.034157-0 21862758

[17] Turgis M, Han J, Caillet S, Lacroix M. Antimicrobial activity of mustard essential oil against *Escherichia coli* O157:H7 and *Salmonella typhi*. Food Control. 2009;20(12): 1073–1079.

[18] Xu H, Dephoure N, Sun H, Zhang H, Fan F, Liu J, et al Proteomic profiling of paclitaxel treated cells identifies a novel mechanism of drug resistance mediated by PDCD4. J Proteome Res. 2015;14(6): 2480–2491. 10.1021/acs.jproteome.5b00004 25928036

[19] Kawatani M, Muroi M, Wada A, Inoue G, Futamura Y, Aono H, et al Proteomic profiling reveals that collismycin A is an iron chelator. Scientific Reports. 2016;6: 38385 10.1038/srep38385 27922079 PMC5138588

[20] Zainal-Abidin Z, Mohd-Said S, Adibah F, Majid A, Mustapha WAW, Jantan I. Anti-bacterial activity of cinnamon oil on oral pathogens. The Open Conference Proceedings Journal. 2013;4: 5.

[21] Kaskatepe B, Kiymaci ME, Simsek D, Erol HB, Erdem SA. Comparison of the contents and antimicrobial activities of commercial and natural cinnamon oils. Indian J Pharm Sci. 2016;78(4): 8.27168676

[22] Silhavy TJ, Kahne D, Walker S. The bacterial cell envelope. Cold Spring Harb Perspect Biol. 2010;2(5): a000414 10.1101/cshperspect.a000414 20452953 PMC2857177

[23] Wu Y, Bai J, Zhong K, Huang Y, Qi H, Jiang Y, et al Antibacterial activity and membrane-disruptive mechanism of 3-p-trans-coumaroyl-2-hydroxyquinic acid, a novel phenolic compound from pine needles of *Cedrus deodara*, against *Staphylococcus aureus*. Molecules. 2016;21(8). 10.3390/molecules21081084 PMC6273998 27548123

[24] Gerding MA, Ogata Y, Pecora ND, Niki H, de Boer PA. The trans-envelope Tol-Pal complex is part of the cell division machinery and required for proper outer-membrane invagination during cell constriction in *E*. *coli*. Mol Microbiol. 2007;63(4): 1008–1025. 10.1111/j.1365-2958.2006.05571.x 17233825 PMC4428343

[25] Ukuku DO, Geveke DJ, Cooke P, Zhang HQ. Membrane damage and viability loss of *Escherichia coli* K-12 in apple juice treated with radio frequency electric field. J Food Prot. 2008;71(4): 684–690. 18468020 10.4315/0362-028x-71.4.684

[26] Shisler KA, Broderick JB. Glycyl radical activating enzymes: structure, mechanism, and substrate interactions. Arch Biochem Biophys. 2014;546: 64–71. 10.1016/j.abb.2014.01.020 24486374 PMC4083501

[27] Wagner AF, Schultz S, Bomke J, Pils T, Lehmann WD, Knappe J. YfiD of *Escherichia coli* and Y06I of bacteriophage T4 as autonomous glycyl radical cofactors reconstituting the catalytic center of oxygen-fragmented pyruvate formate-lyase. Biochem Biophys Res Commun. 2001;285(2): 456–462. 10.1006/bbrc.2001.5186 11444864

[28] Willi J, Kupfer P, Evequoz D, Fernandez G, Katz A, Leumann C, et al Oxidative stress damages rRNA inside the ribosome and differentially affects the catalytic center. Nucleic Acids Res. 2018;46(4): 1945–1957. 10.1093/nar/gkx1308 29309687 PMC5829716

[29] Vogel C, Silva GM, Marcotte EM. Protein expression regulation under oxidative stress. Mol Cell Proteomics. 2011;10(12): M111 009217 10.1074/mcp.M111.009217 21933953 PMC3237073

[30] Bunner AE, Nord S, Wikstrom PM, Williamson JR. The effect of ribosome assembly cofactors on in vitro 30S subunit reconstitution. J Mol Biol. 2010;398(1): 1–7. 10.1016/j.jmb.2010.02.036 20188109 PMC2866118

[31] Nord S, Bylund GO, Lovgren JM, Wikstrom PM. The RimP protein is important for maturation of the 30S ribosomal subunit. J Mol Biol. 2009;386(3): 742–753. 10.1016/j.jmb.2008.12.076 19150615

[32] Cosentino M, Luini A, Bombelli R, Corasaniti MT, Bagetta G, Marino F. The essential oil of bergamot stimulates reactive oxygen species production in human polymorphonuclear leukocytes. Phytother Res. 2014;28(8): 1232–1239. 10.1002/ptr.5121 24458921

[33] Yoo CB, Han KT, Cho KS, Ha J, Park HJ, Nam JH, et al Eugenol isolated from the essential oil of *Eugenia caryophyllata* induces a reactive oxygen species-mediated apoptosis in HL-60 human promyelocytic leukemia cells. Cancer Lett. 2005;225(1): 41–52. 10.1016/j.canlet.2004.11.018 15922856

[34] Carvalho RL, Cabral MF, Germano TA, de Carvalho WM, Brasil IM, Gallão MI, et al Chitosan coating with trans-cinnamaldehyde improves structural integrity and antioxidant metabolism of fresh-cut melon. Postharvest Biol Tech. 2016;113: 29–39.

[35] Ciftci O, Ozdemir I, Tanyildizi S, Yildiz S, Oguzturk H. Antioxidative effects of curcumin, beta-myrcene and 1,8-cineole against 2,3,7,8-tetrachlorodibenzo-p-dioxin-induced oxidative stress in rats liver. Toxicol Ind Health. 2011;27(5): 447–453. 10.1177/0748233710388452 21245202

[36] Bubols GB, Vianna Dda R, Medina-Remon A, von Poser G, Lamuela-Raventos RM, Eifler-Lima VL, et al The antioxidant activity of coumarins and flavonoids. Mini Rev Med Chem. 2013;13(3): 318–334. 22876957 10.2174/138955713804999775

[37] Dahham SS, Tabana YM, Iqbal MA, Ahamed MB, Ezzat MO, Majid AS, et al The anticancer, antioxidant and antimicrobial properties of the sesquiterpene beta-caryophyllene from the essential oil of *Aquilaria crassna*. Molecules. 2015;20(7): 11808–11829. 10.3390/molecules200711808 26132906 PMC6331975

[38] Pavan B, Dalpiaz A, Marani L, Beggiato S, Ferraro L, Canistro D, et al Geraniol pharmacokinetics, bioavailability and its multiple effects on the liver antioxidant and xenobiotic-metabolizing enzymes. Front Pharmacol. 2018;9: 18 10.3389/fphar.2018.00018 29422862 PMC5788896

[39] Rahal A, Kumar A, Singh V, Yadav B, Tiwari R, Chakraborty S, et al Oxidative stress, prooxidants, and antioxidants: the interplay. Biomed Res Int. 2014;2014: 761264 10.1155/2014/761264 24587990 PMC3920909

[40] Mimica-Dukić N, Orčić D, Lesjak M, Šibul F. Essential oils as powerful antioxidants: misconception or scientific fact? Medicinal and aromatic crops: production, phytochemistry, and utilization ACS Symposium Series. 1218: American Chemical Society; 2016 p. 187–208.

[41] Bezerra DP, Militao GCG, de Morais MC, de Sousa DP. The dual antioxidant/prooxidant effect of eugenol and its action in cancer development and treatment. Nutrients. 2017;9(12). 10.3390/nu9121367 PMC5748817 29258206

[42] Cronan JE, Thomas J. Bacterial fatty acid synthesis and its relationships with polyketide synthetic pathways. Methods Enzymol. 2009;459: 395–433. 10.1016/S0076-6879(09)04617-5 19362649 PMC4095770

[43] Broussard TC, Price AE, Laborde SM, Waldrop GL. Complex formation and regulation of *Escherichia coli* acetyl-CoA carboxylase. Biochemistry. 2013;52(19): 3346–3357. 10.1021/bi4000707 23594205

[44] Emiola A, Andrews SS, Heller C, George J. Crosstalk between the lipopolysaccharide and phospholipid pathways during outer membrane biogenesis in *Escherichia coli*. Proc Natl Acad Sci USA. 2016;113(11): 3108–3113. 10.1073/pnas.1521168113 26929331 PMC4801286

[45] Moynié L, Leckie SM, McMahon SA, Duthie FG, Koehnke A, Taylor JW, et al Structural insights into the mechanism and inhibition of the β-hydroxydecanoyl-acyl carrier protein dehydratase from *Pseudomonas aeruginosa*. J Mol Biol. 2013;425(2): 365–377. 10.1016/j.jmb.2012.11.017 23174186

[46] Wendel AA, Lewin TM, Coleman RA. Glycerol-3-phosphate acyltransferases: rate limiting enzymes of triacylglycerol biosynthesis. Biochim Biophys Acta. 2009;1791(6): 501–506. 10.1016/j.bbalip.2008.10.010 19038363 PMC2737689

[47] Yao J, Rock CO. Phosphatidic acid synthesis in bacteria. Biochim Biophys Acta. 2013;1831(3): 495–502. 10.1016/j.bbalip.2012.08.018 22981714 PMC3548993

[48] Birkner JP, Poolman B, Kocer A. Hydrophobic gating of mechanosensitive channel of large conductance evidenced by single-subunit resolution. Proc Natl Acad Sci USA. 2012;109(32): 12944–12949. 10.1073/pnas.1205270109 22826215 PMC3420157

[49] Blount P, Iscla I, Moe PC, Li Y. MscL: The bacterial mechanosensitive channel of large conductance Curr Top Membr. 58: Academic Press; 2007 p. 201–33.

[50] Lovering AL, Safadi SS, Strynadka NC. Structural perspective of peptidoglycan biosynthesis and assembly. Annu Rev Biochem. 2012;81: 451–478. 10.1146/annurev-biochem-061809-112742 22663080

[51] Egan AJ, Jean NL, Koumoutsi A, Bougault CM, Biboy J, Sassine J, et al Outer-membrane lipoprotein LpoB spans the periplasm to stimulate the peptidoglycan synthase PBP1B. Proc Natl Acad Sci USA. 2014;111(22): 8197–8202. 10.1073/pnas.1400376111 24821816 PMC4050580

[52] Zhang G, Meredith TC, Kahne D. On the essentiality of lipopolysaccharide to Gram-negative bacteria. Curr Opin Microbiol. 2013;16(6): 779–785. 10.1016/j.mib.2013.09.007 24148302 PMC3974409

[53] Rosenfeld Y, Shai Y. Lipopolysaccharide (endotoxin)-host defense antibacterial peptides interactions: role in bacterial resistance and prevention of sepsis. Biochim Biophys Acta. 2006;1758(9): 1513–1522. 10.1016/j.bbamem.2006.05.017 16854372

[54] Breazeale SD, Ribeiro AA, Raetz CR. Origin of lipid A species modified with 4-amino-4-deoxy-L-arabinose in polymyxin-resistant mutants of *Escherichia coli*. An aminotransferase (ArnB) that generates UDP-4-deoxyl-L-arabinose. J Biol Chem. 2003;278(27): 24731–24739. 10.1074/jbc.M304043200 12704196

[55] Lee CJ, Liang X, Gopalaswamy R, Najeeb J, Ark ED, Toone EJ, et al Structural basis of the promiscuous inhibitor susceptibility of *Escherichia coli* LpxC. ACS Chem Biol. 2014;9(1): 237–246. 10.1021/cb400067g 24117400 PMC3947053

[56] Barb AW, Zhou P. Mechanism and inhibition of LpxC: an essential zinc-dependent deacetylase of bacterial lipid A synthesis. Curr Pharm Biotechnol. 2008;9(1): 9–15. 18289052 10.2174/138920108783497668 PMC3022321

[57] Itri R, Junqueira HC, Mertins O, Baptista MS. Membrane changes under oxidative stress: the impact of oxidized lipids. Biophys Rev. 2014;6(1): 47–61. 10.1007/s12551-013-0128-9 28509959 PMC5425709

