# Supplementary material for: Disruption of KPC-producing Klebsiella pneumoniae membrane via induction of oxidative stress by cinnamon bark (Cinnamomum verum J. Presl) essential oil
Source: PLoS One. 2019 Apr 2;14(4):e0214326. doi: 10.1371/journal.pone.0214326 (PMC6445408; doi:10.1371/journal.pone.0214326)
Supplement: S1 Table — (DOCX) [file pone.0214326.s003.docx]

**Supporting information**

**Table 1S. List of primers used in this study.**

| Gene | Sequence | Efficiency (%) |
| --- | --- | --- |
| F-*16S rRNA* | CGGCCGGGAACTCAAAGGAG | 91.0 |
| R-*16S rRNA* | TCTCGCGAGGTCGCTTCTCT |  |
| F-*cdd* | GTCATTGCCGTCAGTTTA | 94.4 |
| R-*cdd* | GGTCCTGTTCATCCATCA |  |
| F-*fabA* | CTACCGCATCCACTTCAA | 90.3 |
| R-*fabA* | GAACAGACCCACTTTCAGAT |  |
| F-*thiE* | AATGAACAGTCGGGCGTGAT | 91.4 |
| R-*thiE* | ACCTGATTTTCCACCCGTCC |  |
| F-*udp* | CCTCGGATACCTTCTACC | 93.4 |
| R-*udp* | GGCGGATTCCATTTCATA |  |
| F-*OmpK36* | CGGTTACGGCCAGTGGGAATA | 103.9 |
| R-*OmpK36* | AATTCCGGCAGAACGTCGGTCC |  |

F, forward primer; R, reverse primer.
